# Community health workers in palliative care provision in low-income and middle-income countries: a systematic scoping review of the literature

**DOI:** 10.1136/bmjgh-2020-002368

**Published:** 2020-05-25

**Authors:** MacKenzie Clark MacRae, Owais Fazal, James O'Donovan

**Affiliations:** 1 Department of Medicine, Tufts University School of Medicine, Boston, Massachusetts, USA; 2 Division of Research, Rice University, Houston, Texas, USA; 3 Department of Education, Oxford University, Oxford, UK

**Keywords:** systematic review, public health, health education and promotion, prevention strategies

## Abstract

**Background:**

Community health workers (CHWs) are currently deployed in improving access to palliative care in a limited number of low-income or middle-income countries (LMICs). This review therefore aimed to document evidence from LMICs regarding (1) where and how CHWs are currently deployed in palliative care delivery, (2) the methods used to train and support CHWs in this domain, (3) the evidence surrounding the costs attached with deploying CHWs in palliative care provision and (4) challenges and barriers to this approach.

**Methods:**

We conducted a systematic scoping review of the literature, adhering to established guidelines. 11 major databases were searched for literature published between 1978 and 2019, as well as the grey literature.

**Findings:**

13 original studies were included, all of which were conducted in sub-Saharan African countries (n=10) or in India (n=3). Ten described a role for CHWs in adult palliative care services, while three described paediatric services. Roles for CHWs include raising awareness and identifying individuals requiring palliative care in the community, therapeutic management for pain, holistic home-based care and visitation, and provision of psychological support and spiritual guidance. Reports on training context, duration and outcomes were variable. No studies conducted a formal cost analysis. Challenges to this approach include training design and sustainability; CHW recruitment, retention and support; and stigma surrounding palliative care.

**Conclusion:**

Despite relatively limited existing evidence, CHWs have important roles in the delivery of palliative care services in LMIC settings. There is a need for a greater number of studies from different geographical contexts to further explore the effectiveness of this approach.

**Key questions**

**What is already known?**

- Palliative care is not available for the majority of the world’s population, particularly those who live in low-income or middle-income countries (LMICs).
- Community health workers (CHWs) currently have important roles in the provision of palliative care in LMIC settings and beyond; however, understanding of their roles and effectiveness within the global context is limited.

**What are the new findings?**

- This is the first study to systematically review the evidence across LMICs regarding the role of CHWs in the provision of palliative care services.
- From the 13 studies included in this review, CHWs appear to have a role in raising awareness and identifying individuals requiring palliative care in the community, therapeutic management of pain, holistic home-based care delivery and visitation, and provision of psychological support and spiritual guidance.
- There is a relative lack of studies providing detailed descriptions of CHW training for palliative care provision, as well as the financial implications of such an approach.

**What do the new findings imply?**

- The use of CHWs to assist in palliative care delivery appears acceptable and feasible, although further studies exploring their effectiveness are warranted, given the limited evidence base.
- Further studies evaluating the role of CHWs in palliative care delivery within LMICs across geographically diverse populations with different backgrounds, social values and lifestyles are warranted.

## Introduction

The WHO defines palliative care as the ‘active improvement in quality of life and relief of suffering for patients with incurable disease’.^1^ A subspecialty of palliative care, paediatric palliative care, focuses on the ‘alleviation of suffering and improving the quality of living and dying in children with chronic and life-limiting illnesses’.^2^ Palliative care has proven to be an effective approach for individuals with life-limiting, chronic illnesses and aims to prevent and relieve suffering through the early identification, assessment and impeccable management of pain and other problems by encompassing physical, psychosocial and spiritual support for the terminally ill and their families.^3^ Its delivery should comprise a multidisciplinary approach of continuous and coordinated care involving the family and using community resources wherever a patient’s care takes place, whether in tertiary health centres, community centres or an individual’s home.^2^

Yet, despite being recognised as a human right by the WHO, palliative care is not available for the majority of the world’s population, particularly those who live in low-income or middle-income countries (LMICs).^1^ It is estimated that of the 40 million people worldwide who require palliative care, only 14% receive it, the majority of whom resides in high-income countries (HICs).^2^ This gulf in access has led to palliative care being described as one of the greatest disparities in global healthcare.^4^

The reasons for the lack of palliative care provision in LMICs are complex and multifactorial, and include the lack of access to readily available oral morphine,^5 6^ limited training on palliative care,^7 8^ misconceptions and stigma surrounding opioid analgesia,^9^ and an existing high burden of disease superimposed onto fragile and underfinanced health systems.^10^ Furthermore, the lack of trained health professionals and human resources to deliver palliative care services has also been cited as a key barrier.^11 12^

In order to address the shortage of healthcare professionals capable of providing palliative care services in LMICs, training alternative providers in the community through task-shifting approaches has been suggested.^4^ One such cadre of ‘close-to-the-community workers’ are community health workers (CHWs), individuals working within their own community in a health promotion, prevention and delivery role, who belong to the formal health system but do not have a paraprofessional degree or tertiary education.^13^ CHWs have an essential role in the communities in which they work by providing health services where otherwise none may have been available.^14^ CHWs already have a well-established role in delivering palliative care services in certain LMIC settings. For example, since 1993, Hospice Africa Uganda (HAU) has been providing palliative care services to patients and their families in Uganda. In this model, CHWs (known as community volunteer workers) provide basic nursing care, psychosocial support and, where necessary, refer patients who require palliative care to established hospice services. They also provide holistic care and act as patient advocates.^15^ Similarly, in Rwanda, a multidisciplinary palliative care system is integrated into the public healthcare system. Since 2014, 481 CHWs have provided home-based palliative care services to patients after discharge from the district general hospital, leading to a reduced stress in the continuum of care.^16 17^

The WHO has published guidelines recognising the potential merits of deploying CHWs in the provision of palliative care.^4^ The list of potential CHW functions that have been outlined includes ‘facilitating access to supplies and medicines, routinely conducting assessments of the patient’s physical, psychosocial and spiritual needs, developing a personal care plan in conjunction with family members, providing information and keeping records, and encouraging the family to keep the patient involved in their daily lives as much as possible’.^4^ The World Health Assembly Resolution on Palliative Care in 2014, the first global resolution on palliative care, was signed by 194 United Nations member countries with the intent to reduce barriers to palliative care by integrating it into comprehensive health services throughout the life course.^18^ Therefore, in many countries, CHWs in the community are already providing essential palliative care services for people with serious or life-threatening illnesses; however, little is known outside of the specific places in which they work, about their work.

Although existing reviews assess the provision of palliative care in LMICs more generally,^3 12 19^ no comprehensive review has specifically assessed the available evidence regarding the roles CHWs currently have in palliative care provision. Therefore, we sought evidence from LMIC settings to evaluate four broad areas, including

1. Where and how CHWs are currently deployed in palliative care provision.
2. The methods used to train and support CHWs in the provision of palliative care, including the content, duration and outcomes of training.
3. The evidence surrounding the costs attached with deploying CHWs to assist in the provision of palliative care.
4. Perceived challenges and barriers to CHWs’ involvement in palliative care provision.

## Methods

### Nature of review

We conducted a scoping review to address this broad research topic in an exploratory manner, as well as outline ‘key concepts, types of evidence, and gaps in research’.^20^ We followed Arksey and O’Malley’s widely used methodological framework for conducting scoping reviews to ensure rigour and potential for replicability.^21^

### Search strategy and study selection criteria

The methodology for this scoping review was based on previous reviews conducted by the same lead author in 2018 and 2019.^22 23^ Following a manual search of the Cochrane Library, the Campbell Collaboration, the International Prospective Register of Systematic Reviews and grey literature, we identified no existing or scheduled reviews on the topic of CHWs and palliative care.

We designed a thorough and sensitive search strategy by developing key search terms for ‘community health workers’ and ‘palliative care’ (see online supplementary table 1). Studies were manually filtered at the title and abstract screening stage to include those defined as LMICs using the World Bank Group Classification of Economies.^24^ We filtered results manually in order to identify related studies from HICs to help inform the discussion section of this review.

10.1136/bmjgh-2020-002368.supp1 Supplementary data

We searched the following 11 databases for research published between 12 September 1978 (the date of the Alma Ata Declaration, which declared CHWs as central to primary healthcare)^25^ to 2 July 2019:

- Medline; Embase; Allied and Complementary Medicine Database; and Global Health, via Ovid.
- Cumulative Index to Nursing and Allied Health Literature via Ebsco.
- PsychInfo; Web of Science; Scopus; and Applied Social Sciences Index Abstracts, via ProQuest.
- British Education Index and Education Resources Information Centre.

Please refer to online supplementary table 1 for the detailed search criteria for each database.

We also included additional non-peer-reviewed literature identified through the Electronic Theses Online Service (EThOS), Google Scholar, and websites of research institutions, charities, relevant government departments and international agencies involved in palliative care provision in LMIC settings (eg, HAU). We also conducted a manual search of grey literature databases. Finally, we searched the reference lists of all relevant papers using the snowball sampling technique.

When full texts of potentially relevant studies were not available, we emailed the lead author to request the full text. Authors were given 2 weeks to reply before being sent a further follow-up email. No restrictions were placed on study language.

### Inclusion and exclusion criteria

Studies were included if

1. The primary participants of the study were CHWs. To capture all relevant literature, a combination of over 60 search terms based on CHW descriptions used in a previous systematic reviews was developed.^26–28^
2. CHWs worked in a country defined as an LMIC according to World Bank Group Classification of Economies.^24^
3. The primary aim of the study was to describe or evaluate the role of CHWs in palliative care provision.

Studies were excluded if

1. The primary focus of the study was on health workers other than CHWs. For example, studies which assessed the role of medical professionals, such as doctors, medical students and nurses, or allied healthcare professionals, such as physician assistants or physiotherapists, were excluded.
2. The study was not a full-text, original study; articles such as commentaries, letters, review articles, policy briefs and study protocols were excluded.

Both quantitative and qualitative studies were included.

#### Population

Consistent with recognised definitions, we defined CHWs as health workers who are members of the communities where they work but without formal professional, or paraprofessional, certificated tertiary education.^29^ They should work in the community (rather than a health facility), belong to the formal health system and perform tasks related to healthcare delivery.^29^

#### Intervention

Studies included in this scoping review focus on the role of CHWs in palliative care provision. For the purpose of this review, ‘palliative care’ was used as an umbrella term to define services outside of curative care related to ‘the relief of pain and distressing symptoms or improving the quality of living and dying through meeting physical, social, cultural, spiritual and psychological needs’.^2^ This could also include the terms ‘end-of-life care’ or ‘hospice care’. We included studies concerning the role of CHWs in both adult and paediatric palliative care.

#### Comparator

A comparator was not included.

#### Outcomes

The outcomes for our scoping review were (1) where and how CHWs are currently deployed in palliative care delivery; (2) the methods used to train and support CHWs in the provision of palliative care, including the content, duration and outcomes of training; (3) the evidence surrounding the costs attached with deploying CHWs to assist in the provision of palliative care; (4) perceived challenges and barriers to CHWs’ involvement in palliative care provision. This scoping review is a mapping exercise to address and understand the current existing literature on how CHWs are deployed in palliative care in LMICs.

### Study selection, data extraction and analysis

All studies identified through the initial search of the databases and grey literature were imported into EndNote referencing software V.9. An automated function in EndNote was used to remove duplicate results, and remaining studies were then manually checked by one author (JOD) to ensure all duplicates had been removed. Two authors (JOD and OF) then screened all remaining studies for potential relevance based on the full title and abstract. Where there was a disagreement a third reviewer was consulted to break ties (MM).

At the end of this process the full texts of potentially relevant studies were obtained and independently screened by the three study authors for final inclusion or exclusion against the pre-defined criteria outlined above. Once the final set of studies was agreed on, data were extracted by one author (OF) into a Microsoft Excel version 16.3 spreadsheet for final analysis (see online supplementary material). The use of a data charting form has been recommended as a key stage of conducting a scoping review.^21^ Finally, two authors individually checked the included studies to ensure they agreed with the data which had been charted (JOD and MCM).

### Patient and public involvement

Patients and/or the public were not involved in the design, conduct, reporting or dissemination plans of this research.

## Results

### Search results

The literature search of 11 databases yielded 1797 results, in addition to a further eight studies identified through a combination of a search of the peer-reviewed grey literature and snowball sampling. After duplicates were removed, a total of 1223 results were left for title and abstract screening. This process resulted in 1185 articles being excluded, leaving 35 articles for a full-text review. Following a full-text review, 22 articles were excluded, leaving 13 studies published between 2007 and 2019 for inclusion in the final analysis.

**Figure 1:**
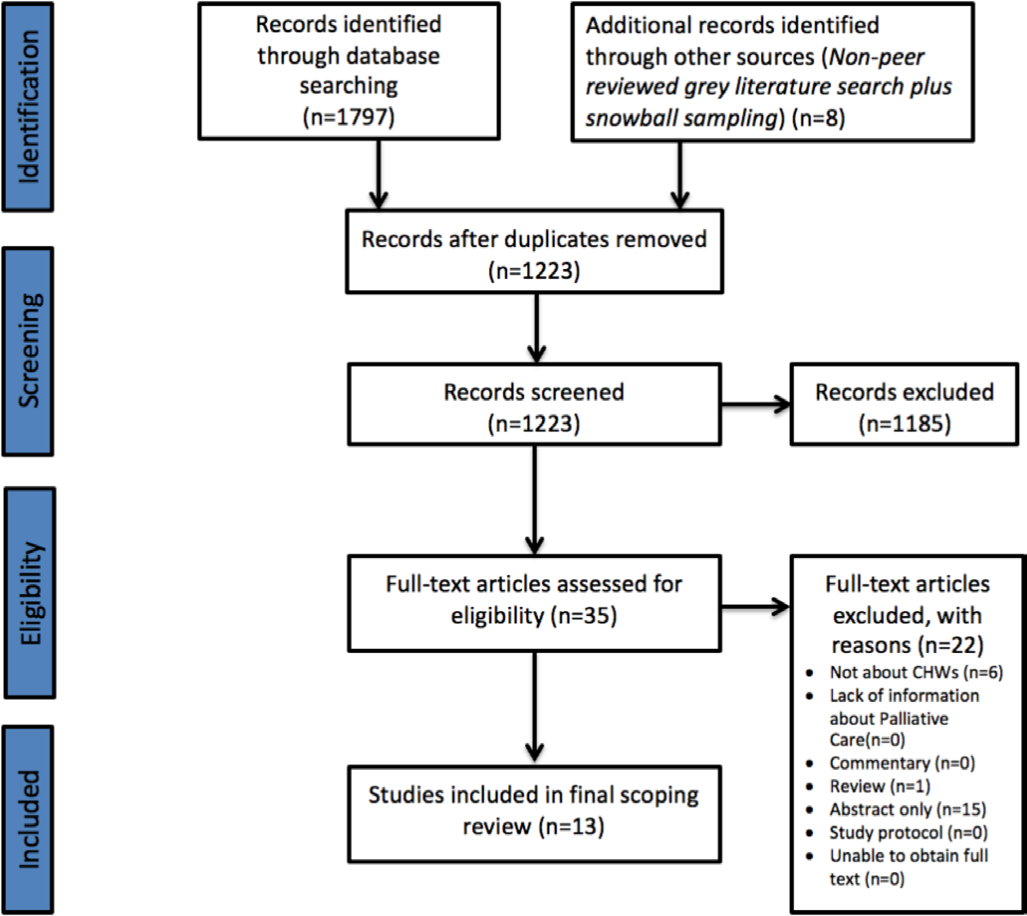
PRISMA diagram. The PRISMA diagram details the search and selection process applied during the scoping review.

For further information, including reasons for study exclusion at the full-text screening stage, please refer to the Preferred Reporting Items for Systematic Reviews and Meta-Analyses diagram (figure 1).

### Study characteristics

**Figure 2:**
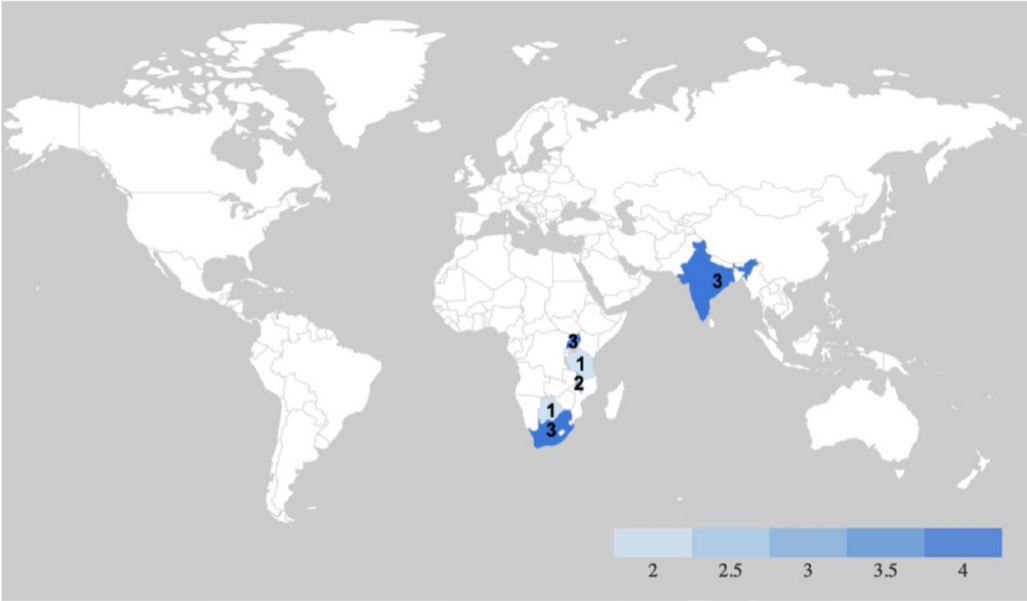
Choropleth map of study locations. A Choropleth Map highlighting the country of origin for each study included in this scoping review.

The majority of studies (n=10) were concentrated in sub-Saharan African countries, including Uganda (n=3),^30–32^ South Africa (n=3),^33–35^ Malawi (n=2),^36 37^ Tanzania (n=1)^38^ and Botswana (n=1).^39^ The remaining studies were all located in India (n=3)^40–42^ (see figure 2).

A total of 15 different terms were used to describe CHWs across the 13 studies. Descriptions of CHWs ranged from those in Uganda trained in carrying out roles specific to palliative care, such as ‘practical, physical, emotional and spiritual care for patients in their own homes’,^32^ to community health volunteers in India who carried out more general roles and historically served as traditional birthing attendants in their villages.^41^

Ten studies described the role of CHWs in adult palliative care services (n=10),^31 32 34 36–42^ and three studies described the role of CHWs in paediatric services (n=3).^30 33 35^

#### Roles of CHWs in palliative care

A range of different roles for CHWs were described across the 13 studies (see online supplementary table 3).

#### Raising awareness and identifying individuals requiring palliative care in the community

In six studies, CHWs played a role in identifying individuals requiring access to palliative care, as well as raising awareness in the community regarding how to access these services (n=6).^30–32 35 41 42^ For example, in Uganda, a non-governmental organisation, HAU, trained CHWs to act as a link between individuals in the community and health centres by finding patients who required the input of the palliative care team. A retrospective, comparative survey comparing the use of HAU services from January to June 2006 and from January to June 2007 showed a 129% increase in the number of new referrals of children due to volunteers finding patients ‘deep in the villages’.^30 31^ Similarly, in India, CHWs in the College of Nursing Community Health programme worked for 2–3 hours per day to identify, provide support to and monitor vulnerable older adults in rural areas.^41^ This resulted in clients and family members receiving assistance in accepting their illness, managing stress and adopting palliative care.^41^

#### Therapeutic management of pain

In six studies, CHWs had a role in therapeutic management of pain (n=6).^30 31 35 38 41 42^ For example, in Uganda, CHWs from HAU assisted in pain control via the provision of oral morphine, as well as ‘first-line chemotherapy’ for 261 children with Burkitt’s lymphoma.^30^ A retrospective, comparative survey comparing the use of HAU services from January to June 2006 and from January to June 2007 showed a 175% increase in the number of children prescribed morphine, and 282% more children completed chemotherapy between the two study periods.^30^ In a cross-sectional, non-interventional interview, 100% of children and caregivers rated the provision of oral morphine and chemotherapy as a strength of HAU.^30^

In a different study from South Africa, 13 CHWs were trained in pain assessment and effective pain management and were provided with basic medicines to use in the care of HIV-positive children at home.^35^ Caregivers appeared to value the home-based model of care, stating, ‘at the hospital a child is not free to talk or ask for anything, home is better’.^35^

#### Holistic home-based care delivery and visitation

Another common role for CHWs was to carry out home-based care and visits for patients with chronic disease and terminal illnesses (n=7).^30–32 34 36–38^ Direct physical care from the CHWs was described in studies in Malawi, Uganda, Tanzania and South Africa.^30–32 35^ For example, in Malawi, CHWs were trained in basic physical therapy skills, including therapy for movement, comfort and recovery. During home visits, CHWs assisted individuals in proper feeding positions, cane walking, side lying for comfort and standing up from a chair.^36 37^ Patient reports indicated that CHWs allowed them to become ‘more independent, participatory and productive in their communities’.^37^ In Tanzania, the roles of CHWs were cleaning the individuals homes (65%), washing individuals (53%), dressing individuals (47%) and preparing meals (38%), as well as reporting the individual’s clinical condition to healthcare officers at the hospital and assisting with medications.^38^

#### Psychological support and spiritual guidance

In seven studies, CHWs were involved in psychological support and spiritual guidance of both caregivers and individuals (n=7).^31–33 35 40–42^ For example, CHWs in India reported once ‘physical problems of the terminally-ill individuals were out of the way, emotional support was what I provided the’.^40^ In a study from South Africa by Naicker *et al*,^35^ CHWs encouraged caregivers of HIV-positive children to regularly play with them. This served to support the social and emotional well-being of the child beyond their physical needs.^35^ Additionally, the authors of this study integrated spiritual aspects, such as prayer and song, into the CHW training sessions so they could address the spiritual needs of families, caregivers and children.^35^ Further, in a study by Amery *et al*,^30^ 80% of the 11 children in the cancer ward and at HAU rated volunteer-led play services as a service strength with caregivers stating, ‘when he plays he feels comfortable and forgets all about the disease’.^30^

### Description of CHW training

Ten studies provided details regarding how CHWs were trained in the provision of palliative care (n=10).^31–37 40–42^ However, there was significant heterogeneity across studies, making direct comparisons challenging.

Five of the CHW training programmes included in this review were described as 7 days or less in duration (n=5),^31 32 34 40 42^ while Nesbit *et al*^36 37^ conducted training over a 3-week period. The majority of CHW trainings were one-off programmes (n=7),^33–37 40 42^ but several studies included details surrounding ongoing education and support for CHWs in the form of monthly meetings and day-to-day advice provided remotely through mobile phone support (n=3).^31 32 41^

Within the training programmes, there was variability in content determined by the goals of each study. For example, studies by Jack *et al*^31 32^ provided significant details surrounding their comprehensive training course taught by a member of the hospice team at HAU over a 6-day period. The course content ranged from an overview of the fundamentals of palliative care and home nursing care to practical sessions on communication skills and bereavement support. In contrast, studies by Nesbit *et al*^36 37^ focused on CHW training in a specific skillset within rehabilitation and physical therapy.

There was variability across studies regarding training methods, course leaders and materials. CHW trainings ranged from didactic classroom learning^31 32^ to mentorship between participants,^33^ informal shadowing in health centres^40^ and dissemination of information through support packages, with illustrated handouts and guides for home-based care.^35^ Several studies involved engagement from multiple stakeholders, including palliative care doctors, nurses and physical therapists,^36 37 41^ whereas others were led solely by nurses.^31 32^

Details on CHW evaluation were poorly documented across the studies included in this review, with only five studies providing details on how CHWs were assessed following training. Evaluation ranged from post-training evaluative questionnaires^34 36 37^ to evaluative checklists used during observational visits with the CHW,^35^ or in one study through supervision of CHWs during a period of four clinical days.^42^ Where evaluation was documented, the majority of studies reported a one-off assessment of CHWs (n=3),^34 35 42^ while Nesbit *et al*^36 37^ conducted multiple choice preknowledge and postknowledge tests with ongoing formative assessments, continued observation of CHWs during home visits and skills competency checks over a 2-week period.

Three out of the 13 studies included in this review contained no information on the content or duration of training, how training was developed, methods of training evaluation or details regarding ongoing supervision.^30 38 39^

### Financial costs and considerations

No studies conducted a formal cost analysis regarding the use of CHWs in palliative care provision; however, three studies outlined programme costs.^30 31 38^

Pöyhiä *et al*^38^ provided information on the cost of CHW wages and compensation of travel expenses for 16 volunteers in Tanzania. The maximum monetary support was 25 000 Tanzanian shillings (US$11) per month to cover wages and travel expenses, in additional to the provision of a bicycle.^38^

In Uganda, CHWs were provided a bicycle and a T-shirt.^31^ The authors estimated that the annual amount per CHW for bicycle maintenance, uniforms, stationery and supplies for their kits (soap and gloves) was 287 000 Ugandan shillings (US$148),^31^ equivalent to US$12.30 per month.

Amery *et al*^30^ reported that the total annual programme costs for HAU was £27 657.55 (US$36 875.81). In this study, it was estimated that the average cost per child receiving palliative care services in Uganda at HAU is US$75^30^; however, a formal cost analysis was not conducted, and disaggregated costs for CHW deployment were not provided.^30^

### Challenges and barriers to CHW involvement in palliative care provision

Several challenges and barriers were identified across studies regarding the deployment of CHWs in the provision of palliative care.

A lack of training specific to palliative care was identified as problematic.^33 40^ In India, CHWs reported a lack of knowledge in managing emotional distress at the end of life, as well as challenges communicating information with patients and families.^40^ This led to frustration among patients and family members who expected CHWs to handle emergency care needs.^40^ Issues around the sustainability of training programmes was also highlighted in a study from South Africa.^34^ As a result, CHWs suggested a ‘train-the-trainer’ model so that they could educate future CHWs and promote sustainable, self-led access to updated palliative care content.^34^

Other studies identified challenges surrounding CHW recruitment and retention. For example, Jack *et al*^31^ noted that in the first intake of CHWs for the HAU programme in Uganda, only 9 of the original 40 volunteers remained active after 3 years. The CHWs in this particular study cited the cost of travelling to patients and the hospice centre as reasons for the high turnover.^31^ Further, the lack of CHWs from remote areas led to difficulties in dealing with multiple languages and dialects spoken, which hindered communication between patients and the HAU team.^31^ Other studies cited care fatigue among the CHWs as an additional challenge.^39 41^ Several studies reported that dealing with the loss of a patient negatively impacted CHWs and interfered with the quality of care delivered.^33 38–40^ In a study by Kang’ethe,^39^ the author reported that three-quarters of CHWs had not been given adequate psychological support. This was demonstrated by CHWs ‘breaking down in tears’ when explaining the environments that they worked in.^39^

The third set of challenges included general cultural stigmas. In a study by Kang’ethe,^39^ CHWs faced stigma and discrimination in providing palliative care to clients with AIDS, leading the caregivers and patients to seek assistance from traditional healers. This proved challenging, as patients often failed to maintain their medication regimen.^39^

## Discussion

This scoping review captures the variety of roles CHWs currently have in the provision of palliative care across six LMICs. CHW roles include raising awareness and identifying individuals requiring palliative care in the community; therapeutic management of pain; holistic home-based care delivery and visitation; and provision of psychological support and spiritual guidance. Descriptions of CHW training and evaluation were variable, and there was a paucity of evidence regarding the financial implications of deploying CHWs in palliative care provision. Several challenges were identified, including issues with CHW training, recruitment, retention and psychological support, as well as issues of stigma surrounding palliative care delivery.

### Roles for CHWs in the delivery of palliative care in LMIC settings

**Figure 3:**
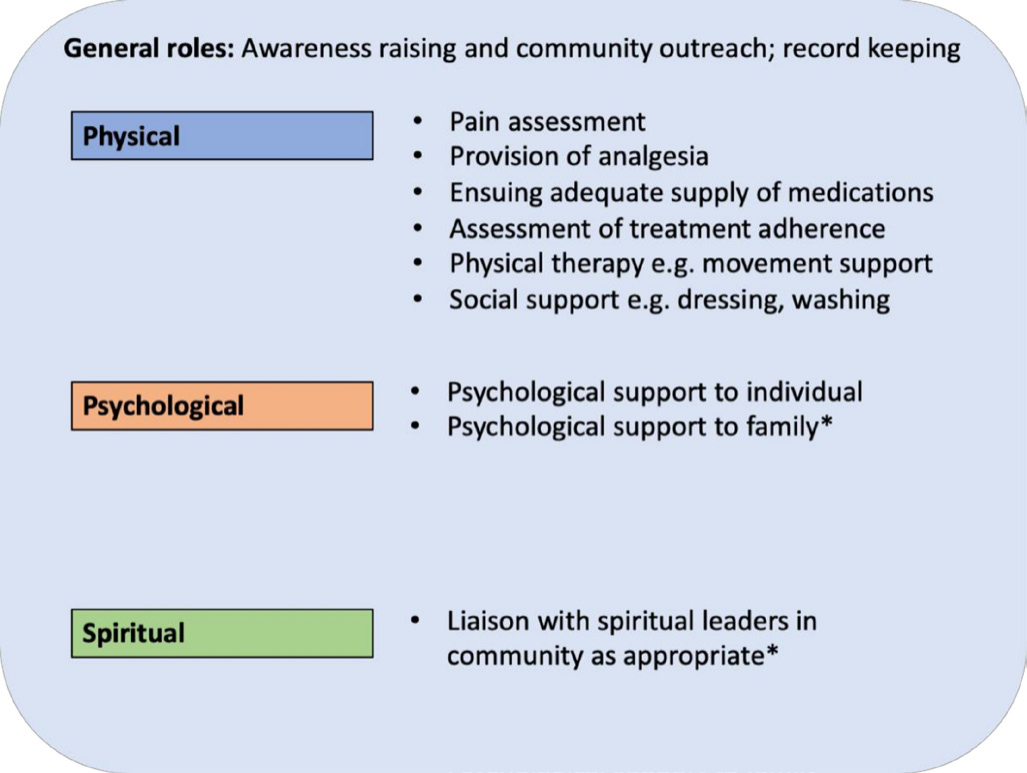
Suggested roles for CHWs in the delivery of palliative care services.

From the existing literature, it is clear that CHWs have several important roles to play in the delivery of palliative care in LMICs, especially in settings where palliative care systems are established. Using this evidence, we have developed a model for the current, existing roles, mapped against the three cornerstones of palliative care: the physical, psychological and spiritual needs of individuals and their families (figure 3). Of note, the roles of CHWs across different contexts vary according to the level of hospice–palliative care development denoted on a global classification scale from countries with no known hospice–palliative care activity, such as Senegal and Liberia (group 1), to countries such as Uganda, South Africa, Malawi and Tanzania, where hospice–palliative care is integrated into mainstream healthcare (group 4).^2^

#### Physical

One role for CHWs could be in the assessment and management of the physical needs of individuals in the community, such as supplying analgesia for pain relief and the provision of physical therapy. With appropriate supervision and training and where analgesia is available, CHWs could be supplied with oral analgesia for distribution during home visits. This model could help to improve access to analgesia, as well as reduce some of the costs encountered by individuals and their families trying to access medicines. For example, in several LMIC settings, the cost of travelling to receive palliative care and analgesia can result in high out-of-pocket payments and the sale of physical assets, such as gold or the family home.^19 43 44^ The sale of such assets can have long-term consequences on the future financial security of families. In other contexts, the model of supplying CHWs with essential diagnostic equipment and medicines to deliver direct home care in the community has had success in reducing under-5 mortality rates through improved medicine access.^45^

Beyond assessment of pain and distribution of analgesia, CHWs have also been identified to have a role in other forms of physical support; however, such extended roles would need to be balanced with the wishes of the patients and their family, as well as the demands of the existing workload of the CHW.

#### Psychological

A second important area for CHWs could be in the provision of psychological support to the patient and their families. Although this was not well-explored in the studies included in this review, roles for CHWs in this domain could include listening to individuals and exploring their concerns. CHWs will require specific training in how to perform this role, and a recent review of psychological interventions in palliative care suggested that cognitive behavioural-based interventions, mindfulness-based interventions and meaning-based interventions could have an important role to play in reducing depression and anxiety among individuals receiving palliative care.^46^

It will also be important for CHWs to be trained in how to provide psychological support and guidance to family members who are often responsible for conducting the mainstay of care. Several studies have demonstrated that caregivers of individuals receiving palliative care can suffer from burnout, depression and fatigue. A recent review suggested that despite limited evidence, psychoeducational support interventions might be beneficial for family carers; however, it should be noted that evidence for the effectiveness of these interventions in LMIC settings is lacking.^47^

As noted in the study by Kang’ethe,^39^ appropriate ongoing support to help CHWs deal with the challenges of addressing complex and difficult aspects of end-of-life care is vital. CHWs themselves must be offered counselling and supported emotionally, including preintervention and regular debriefing sessions, in order to deal with the challenges of providing palliative care and end-of-life support.

#### Spiritual

A third domain is addressing the spiritual needs of individuals receiving palliative care. From this existing literature, this was an underexplored area; however, potential roles could include liaising with spiritual and religious leaders to ensure they are aware of individuals who require specific support, and taking a multidisciplinary approach to care by leveraging existing community support. Such roles would need to be contextually appropriate and tailored to the needs of the individual. Some individuals may have significant spiritual needs, others none.

The specific roles of CHWs outlined here will be dependent on a contextual needs assessment based on the setting where they are being deployed, taking into consideration sociocultural variances and resource availability. Emphasis should also be placed on the importance of a community-based model of care in order to reach the highest number of people, especially those in rural or hard-to-access areas. Such an approach has also been proposed by the American Society of Clinical Oncology in their Resource-Stratified Practice Guidelines.^48^

It is also important to note that, although the mainstay of CHWs work is likely to be in addressing the burden of adult palliative care, the WHO has also highlighted the importance of children’s palliative care in LMICs.^49^ Nearly 2.5 million children die each year with serious health-related suffering, more than 98% of whom reside in LMICs.^50^ Several studies in this review identified the benefits of specialised paediatric palliative care, including increased referrals and service use, improved drug interventions and better therapeutic compliance.^30^ It is therefore important that CHWs are, at a very minimum, made aware of the palliative care options for children who require such services. However, a much larger body of evidence is needed to address specific strategies for paediatric pain management, psychotherapeutic skills to communicate with children, and methods of providing family support.

### Considerations for CHW training

We identified a relative lack of detail across the studies included in this review surrounding design, delivery and evaluation of training for CHWs in the provision of palliative care.

An important consideration for developing the content of CHW training programmes is the need to address the complex psychosocial issues, belief systems and rituals surrounding death and dying. In the study by Kang’ethe,^39^ CHWs reported challenges of clients and caregivers visiting traditional healers. Since traditional healers play a role in the death and bereavement process in some cultures,^51^ CHWs should be trained to work synergistically with them to optimise the spiritual needs and cultural practices of each individual. It is also important to note the benefit of longitudinal support and education to understand how CHWs are sustaining their work and implementing their care following training.^37^ This may encompass ongoing training, which could be supported remotely through the use of mobile phones, which was documented in several studies included in this review.

More attention should be given towards how CHW training is evaluated in this field. It is important to note that one-off assessments using checklists and pretest and post-test assessments of knowledge may not accurately assess the emotional and spiritual skills required of CHWs to deliver effective palliative care. Further, these assessments may not provide insight into the experiences of the CHW during training nor reflect how CHWs will enact their role in the communities.^52^ Taking a more comprehensive approach towards training evaluation is therefore important to capture the holistic nature of skills required by those delivering palliative care. This could include on-the-job assessments of encounters in the community, which would also be more reflective of real-world practice, as demonstrated in the studies by Nesbit *et al.*^36 37^

Strategies focused on long-term motivation and retention of CHWs, such as distribution of materials, supplies and financial incentives, must be considered during the initial training and ongoing assessment of CHWs.^53 54^ Given that the mode of engagement of CHWs as volunteers or employees may affect their long-term motivation and effectiveness in their role, it is essential that CHW programmes consider the implications of these measures when designing training programmes.

### System-level challenges and barriers to deploying CHWs to assist in palliative care provision

The issues highlighted in this review must be contextualised by highlighting the broader systemic issues at hand, including the lack of oral morphine availability worldwide.^55^ The 2017 Lancet Commission on Palliative Care and Pain Relief highlighted the shocking disparities in global morphine-equivalent opioid distribution, and that of the 298·5 million metric tons distributed each year, only 0.1 metric ton was to low-income countries.^55^ The ability of CHWs to perform their roles well is therefore contingent on the support they receive from the health system in which they are embedded, and the resources available to them. At the government level, there are many reasons that account for the inadequate availability of analgesia from low social and economic development to overly restrictive laws and regulations that impede medical access.^56^ Considering these barriers, more general enabling factors are important if CHWs are to play an effective role in the provision of palliative care in LMIC settings. Given that increasing access to analgesia at a system level is a complex, multifactorial intervention,^57 58^ further studies are warranted to better understand and address the contextual processes at play that may otherwise undermine the effectiveness of these interventions.

Strengthening palliative care policies within a country is an important starting point. Lessons can be learnt from pioneering programmes which already exist, such as the Neighbourhood Network in Palliative Care (NNPC) in Kerala, India. The NNPC has made effective use of volunteer CHWs through direct ownership at the community level, in addition to parallel monetary support from the local and state governments.^59^ Through using CHWs, the ‘bottom-up’ development of NNPC has paved the way for community-level initiatives in palliative medicine.^2 59^ This highly effective model leverages existing community-based health systems and is strongly supported at the government level. This support at government level is important. Out of 53 African countries, only four have integrated palliative care into health policy (South Africa, Tanzania, Kenya and Uganda), and two more have stand-alone national palliative care policies (Rwanda and Swaziland).^60^ Such policies should outline clear roles for health workers, such as CHWs, including how they might be appropriately supported and funded. Further, in countries where limited palliative care services exist, CHWs could play a role in the development of services through a participatory design process. This could be one way of ensuring a culturally appropriate programme is developed that is both acceptable and accessible to those who need it most.

Given the complete absence of a discussion around the costs of deploying CHWs to assist in palliative care provision, formal cost evaluations are also required to understand the financial implications of training and deploying CHWs in such roles. In the early stages of deploying CHWs in palliative care, it is essential to consider their effectiveness in delivering palliative care service, prior to cost-effectiveness of such programmes. However, since only 0.15% of research funding globally goes towards palliative care,^61^ it is important to understand how CHW-led initiatives might impact this already under-resourced area, and how health budgets could be adapted to fully support CHWs in this role should they prove to be effective agents of delivering palliative care services.

### Limitations

Regarding study limitations, only 13 studies were identified for inclusion, resulting in a limited evidence base. We are also aware of ongoing or existing initiatives involving CHWs in the provision of palliative care in LMIC settings which have not been formally appraised and documented and thus were not captured in this review. Additionally, we are aware of short reports that exist on the role of CHWs in palliative care in LMIC settings,^62^ which were not included in our review since we chose to include only original research studies with the hypothesis that original research studies would contain sufficient information to draw meaningful conclusions. It is important to note that the studies included within this review originate from a limited number of countries and that there is a distinct lack of studies from countries with limited palliative care services. This could be because CHWs have yet to be integrated and formally recognised in these settings, resulting in a relatively narrow current evidence base. Future studies are therefore needed to explore the role of and effectiveness of CHWs in palliative care in other LMICs.

Further, we did not conduct a quality assessment of the studies included in this final review; however, as noted in the Methods section, this is in keeping with guidelines for conducting a scoping review.^21^ Moreover, there was a heterogeneity of studies included in this review in terms of scope and methodology. We are aware that there are existing studies concerning the roles of volunteers in the delivery of palliative care services in LMIC settings that we have not included in this review due to our definition of CHWs being health workers based in the community (rather than at health facilities) and who do not have a formal professional or paraprofessional certification.^29^ For example, in a study by Deodhar and Muckaden, the educational needs of 14 volunteers in India working to deliver palliative care services were assessed through the use of a questionnaire. However, this study was not included since two of these volunteers held professional degrees, and all of the volunteers were based at a tertiary care oncology institute (rather than in the community).^63^ It is therefore important to note that the literature pertaining to this subject is contested, reflecting wider debates across the field as to who is deemed to be a CHW, a topic that has been keenly debated in recent years.^28 64^ Many of the studies evaluated the outcomes of entire programmes in which CHWs played a support role rather than specifically evaluating the disaggregated effect of CHWs roles. Future studies which explore the role of CHWs in different LMIC settings are needed in order to expand the evidence base regarding the role and effectiveness of CHWs.

## Conclusion

The 13 studies included in this review all took place in sub-Saharan African countries or in India. Despite the limited evidence base, we found that CHWs have several roles in palliative care provision in LMIC settings. These include raising awareness in the community, provision of pain management services, home-based care delivery and visitation, and provision of psychological support and spiritual guidance. Several areas of weakness were also identified, including a lack of details surrounding the training and ongoing support of CHWs, as well as the financial costs associated with deploying CHWs in the provision of palliative care services.

There is a need for countries to develop their own specific guidelines for the roles CHWs should play in palliative care, so that any initiatives are appropriate for the context in which they are being implemented. Finally, it is important to emphasise that CHWs cannot function in isolation to address the burden of palliative care needs in LMICs. Key stakeholders from various domains, including local government and policymakers, must value, advocate for and work in conjunction with CHWs to ensure palliative care, which is a basic human right, can be accessed by all.
