# Supplementary material for: Community health workers in palliative care provision in low-income and middle-income countries: a systematic scoping review of the literature
Source: BMJ Glob Health. 2020 May 25;5(5):e002368. doi: 10.1136/bmjgh-2020-002368 (PMC7252978; doi:10.1136/bmjgh-2020-002368)
Supplement: Supplementary data [file bmjgh-2020-002368supp001.pdf]

Supplementary Material

Table 1. Database search strategies, including search terms

**Database**	**Search strategy employed**
**AMED (Allied and Complementary Medicine) 1985 to August 2019, Embase 1974 to present, Global Health 1973 to 2019 Week 37, Medline 1946 to present, PsycINFO 1806 to present**	(((community adj1 health* adj3 (assistant or advocate or agent or promoter* or worker* or volunteer or aide* or practitioner* or supporter or representative)) or (village adj1 health* adj3 (worker* or team* or guide* or promoter)) or (Accompagnateurs or Activista or Agente comunitario de salud or Agente comunitario de saude or Anganwadi or Animatrice or accredited social health activist* or adherence support worker* or barangay health worker or bare foot doctor or behvarz or brigadista or Colaborador voluntario or doula or female community health volunteer or frontline primary healthcare worker or female health worker or female community health volunteer or gramsakhi or health extension worker* or health activist* or kader or manzaneras or lay health worker or lay counselor or lady health worker* or lady health visitor or lady health worker* or maternal health worker or Monitora or peer educator or promotera or Raedat or rural health assistant* or Saksham sahaya or Sevika or shasthy? shebika* or shasthy? kormi* or trained birth assistant* or Visitadora or voluntary healthworker) or ((community adj3 treatment support*) or mobilizer or nutrition worker or resource person or reproductive health worker or volunteer))<br><br>AND<br><br>(palliative or end-of-life care or terminally ill or hospice or supportive care)).ab.

**SCOPUS**	TITLE-ABS-KEY ("Accompagnateurs" OR "Activista" OR "Agente comunitario de salud" OR "Agente comunitario de saude" OR "Anganwadi" OR "Animatrice" OR "accredited social health activist*" OR "adherence support worker*" OR "barangay health worker" OR "bare foot doctor" OR "behvarz" OR "brigadista" OR "Colaborador voluntario" OR “doulas” OR "Female community health volunteer" OR "female health worker" OR "gramsakhi" OR "health extension worker" OR "health activist" OR "kader" OR "manzaneras" OR "lay health worker" OR "lay counsellor" OR "lady health visitor" OR "lady health worker*" OR "maternal health worker" OR "Monitora" OR "peer educator" OR "promotera" OR "Raedat" OR "rural health assistant*" OR "Saksham sahaya" OR "Sevika" OR "shasthy? shebika*" OR "shasthy? kormi*" OR "trained birth assistant" OR "Visitadora" OR ("community health*" PRE/3 (worker* OR volunteer OR aide* OR practitioner* OR supporter)) OR ("village health*" PRE/3 (worker* OR team* OR guide*)) OR (front?line AND primary AND health?care) OR (community PRE/3 "treatment support*")) OR (community* PRE/3 (distributor* OR "care worker*"))<br><br>AND<br><br>("Palliative” OR “End-of-life care” OR “Terminally ill” OR “Hospice” OR “Supportive care”)
**BEI, ERIC**	(((community adj1 health* adj3 (assistant or advocate or agent or promoter* or worker* or volunteer or aide* or practitioner* or supporter or representative)) or (village adj1 health* adj3 (worker* or team* or guide* or promoter)) or (Accompagnateurs or Activista or Agente comunitario de salud or Agente comunitario de saude or Anganwadi or Animatrice or accredited social health activist* or adherence support worker* or

	barangay health worker or bare foot doctor or behvarz or brigadista or Colaborador voluntario or doula or female community health volunteer or frontline primary healthcare worker or female health worker or female community health volunteer or gramsakhi or health extension worker* or health activist* or kader or manzaneras or lay health worker or lay counselor or lady health worker* or lady health visitor or lady health worker* or maternal health worker or Monitora or peer educator or promotera or Raedat or rural health assistant* or Saksham sahaya or Sevika or shasthy? shebika* or shasthy? kormi* or trained birth assistant* or Visitadora or voluntary healthworker) or ((community adj3 treatment support*) or mobilizer or nutrition worker or resource person or reproductive health worker or volunteer))<br><br>AND<br><br>(palliative or end-of-life care or terminally ill or hospice or supportive care)).ab.
**Cochrane Library**	1. (community adj1 health* adj3 (assistant or advocate or agent or promoter* or worker* or volunteer or aide* or practitioner* or supporter or representative))<br>2. (village adj1 health* adj3 (worker* or team* or guide* or promoter))<br>3. (Accompagnateurs or Activista or Agente comunitario de salud or Agente comunitario de saude or Anganwadi or Animatrice or accredited social health activist* or adherence support worker* or barangay health worker or bare foot doctor or behvarz or brigadista or Colaborador voluntario or doula or female community health volunteer or frontline primary healthcare worker or female health worker or female community health volunteer or gramsakhi or health extension worker* or health activist* or kader or manzaneras or lay health worker or lay counselor or lady health

	worker* or lady health visitor or lady health worker* or maternal health worker or Monitora or peer educator or promotera or Raedat or rural health assistant* or Saksham sahaya or Sevika or shasthy? shebika* or shasthy? kormi* or trained birth assistant* or Visitadora or voluntary healthworker)<br>4. (community adj3 treatment support* or mobilizer or nutrition worker or resource person or reproductive health worker or volunteer)<br>5. (palliative or end-of-life care or terminally ill or hospice or supportive care)<br>6. 1 OR 2 OR 3 OR 4 AND 5

Legend. Search terms for each database searched.

Table 2. Results from individual database searches.

**Database**	**Number of Hits**
**Medline, Embase, Global Health, PsychInfo and AMED via (OVID)**	1,111
**SCOPUS**	252
**BEI via EBSCO**	57
**ERIC via EBSCO**	313
**Cochrane Library**	64
**Grey literature and snowballing**	8

Legend. The results of individual database searches, including the number of hits and the supplementary material page numbers where the search strategies may be found.

Table 3. Summary table of included studies.

Author	Study Title	Year	Country and region	World Health Assembly Mapping Levels of Palliative Care Development (Group 1 'No activity' to Group 4b 'Advanced Integration')	Name given to CHW, number involved in study and cadre description	Purpose and duration of study, role of CHW relevant to delivery of palliative care	Training and Supervision	Results/Key findings	Financial details or Cost Analysis
Amery J.M. et al.	The beginnings of children's palliative care in Africa: evaluation of a children's palliative care service in Africa.	2009	Uganda, East Africa	4b	**Name:** Community Health Volunteers<br><br>**Number:** No details.<br><br>**Description:** No cadre description.<br><br>**Remuneration:** Total staff costs (including transport and other associated expenses for community health volunteers) £5,405.41	**Purpose:** To evaluate a children's palliative care service designed specifically for a resource-poor sub-Saharan African setting.<br><br>**Duration:** 12 months<br><br>**Role of CHW:** The role of CHWs includes ensuring adherence to treatment, screening in villages and local communities for individuals potentially in need of PC, and providing home-care services to terminally ill patients who lack family or community support.	**Content:** No details.<br><br>**Duration:** No details.<br><br>**Person or group responsible for delivering training:**<br><br>**Theories used:** No details.<br><br>**Assessment of training:** No details.<br><br>**Supervision:** No details.	The evaluation suggests that effective nurse-led, volunteer-supported children's palliative care services are achievable in a sub-Saharan African setting. The large increases in children both referred and accepted onto the programme suggest that the service was effective at identifying children in need of palliative care who were not previously being identified or admitted.	Service costs: Total staff costs (including transport and other associated expenses for community health volunteers) £5,405.41. Basic patient needs (food, blankets, transport) £5,584.03. Drugs and pharmacy consumables £8,117.46. General Support and running costs £7,426.00. Transport £1,114.65. Total service cost per year £27,657.55. Average cost per child £50.28 ($US 75).

Visagie B.B. et al.	Needs and challenges of lay community health workers in a palliative care environment for orphans and vulnerable children	2017	South Africa, Africa	4a	**Name:** Lay Community Health Workers<br><br>**Number:** 46<br><br>**Description:** Peer Counsellors, VCT Coordinators, VCT Counsellors, Adherence Counsellors, Day Care Workers, Home Base Carers who were hired by the Home of Hope organization to provide pallaitive care services.<br><br>**Remuneration:** No details.	**Purpose:** The focus of this study is to explore the role of lay community health workers (LCHWs) in a community organization located in rural Bronkhorstspruit, Gauteng Province of South Africa that provides palliative care for orphans and vulnerable children diagnosed with HIV/AIDS. This study also examined what is expected from LCHWs in terms of competencies, abilities and their coping mechanisms, based on their regular interaction with terminally ill and vulnerable children.<br><br>**Duration:** 8 months<br><br>**Role of CHW:** Psycho-social support, such as lay-counseling, for orphans and vulnerable children covered a large area of the LCHWs daily responsibilities.	**Content:** Participants mentored each other in a lay fashion, they followed an informal and unstructured mentoring practice. This mentoring process made it possible for them to learn new knowledge, skills and tasks. In addition to mentoring, the participants also used coaching as a training and learning method and as a way of transferring knowledge and skills about their tasks to each other.<br><br>**Duration:** No details.<br><br>**Person or group responsible for delivering training:** No details.<br><br>**Theories used:** No details.<br><br>**Assessment of training:** No details.<br><br>**Supervision:** No details.	Three main themes emerged from the data analysis: (i) Knowledge needed by LCHWs (ii) skills needed by LCHWs, and (iii) organizational challenges. Specific skills needed by the participants to work competently consisted of: nutrition, academic skills in reading, writing, numeracy and literacy, auxiliary health care, mentoring and coaching. Through the findings, a framework for enhancing the work experiences of the lay community health workers was developed. The uniqueness of this framework is that the focus is on improving the work lives of the lay community health workers, who have serious skills-resourcing needs.	No details

Campbell C. et al.	Community health workers' palliative care learning needs and training: Results from a partnership between a US university and a rural community organization in Mpumalanga province, South Africa.	2016	South Africa, Africa	4a	**Name:** Community Health Workers (CHWs)<br><br>**Number:** 29<br><br>**Description:** CHWs were laypeople trained to work with HIV/ AIDS patients in their respective communities.<br><br>**Remuneration:** No details.	**Purpose:** Three aims: (1) to identify palliative care learning needs of community health workers (CHWs) working in a non-governmental organization (NGO) in Mpumalanga province SA, (2) to develop a training session based on the needs identified by the CHWs, and (3) to describe CHWs' perceptions of the usefulness of the training content.<br><br>**Duration:** No details.<br><br>**Role of CHW:** Currently CHWs are providing care for patients with advanced stages of disease, including HIV/ AIDS, TB, and other chronic illnesses.	**Content:** No details.<br><br>**Person or group responsible for delivering training:** NGO and USAID<br><br>**Theories used:** No details.<br><br>**Assessment of training:** Questionnaire with the following questions: 1. Describe the work that you do. 2. As you think back on your work, tell us about the training/ education that really helped you. 3. What training/ education did you need and not have? 4. Tell me a story about a challenging day at work."<br><br>**Duration:** Half day<br><br>**Supervision:** No details.	Educational sessions were planned and implemented for three of the needs: HIV/ AIDS, palliative care, and debriefing of CHWs.The majority of the CHWs reported they learned something new (mean score of 4.83) and could use the content in their daily work (mean score of 4.70). The remaining evaluation scores were as follows: I feel better able to help my clients (mean score of 4.73) and with this new information I will do things differently (mean score of 4.40) (see Table 2). Additionally the faculty noted that the CHWs were very engaged in the training sessions, participated in role plays, shared stories, and discussed how best to use the material presented.	No details

Jack B.A. et al.	'A bridge to the hospice': the impact of a Community Volunteer Programme in Uganda.	2011	Uganda, East Africa	4b	**Name:** Community Volunteer Workers (CVWs)<br><br>**Number:** 32<br><br>**Description:** Members of the local community who speak the local dialect. The CVWs are selected by senior members of the local community and meet the following criteria: completed a basic education, identified as being respected and trustworthy, and available to volunteer for about 6 hours a week.<br><br>**Remuneration:** No renumeration**.** Cost for meeting attendance reimbursed.	**Purpose:** The aim of this qualitative study was to evaluate the impact of Hopsice Africa Uganda's Community Volunteer Programme, which trains volunteers to provide support to patients in their own homes.<br><br>**Duration:** No details.<br><br>**Role of CHW:** The role of the CVW is to identify and care for people needing palliative care in their own homes, offering support and advice to patients and their families. Roles expected of CHVs include: Identify and referral of patients who need palliative care, educate carers and patients in care and well-being, including nutrition, hygiene, infection control, provide social support to the family and patient and bereavement support, patient and family care regarding management of common illnesses,	**Content:** CVWs undergo a nonresidential course that covers an overview of the fundamentals of palliative care, practical aspects of home nursing care, communication skills, end-of-life care, emotional support for carers, bereavement support and ethics of palliative care.<br><br>**Duration:** 6 days.<br><br>**Person or group responsible for delivering training:** No details.<br><br>**Theories used:** No details<br><br>**Assessment of training:** No details<br><br>**Supervision:** Ongoing education and support are provided by the Hospice team, with monthly meetings being held, and contact via mobile phone for day-to-day support and advice.	Key themes regarding the impact of CVWs included the impact on the patients and their families; physical care, practical help, counselling/education and how they acted as a 'bridge to the hospice'. . All respondents in the study indicated that the Community Volunteer Programme is having a positive impact on patients and their families. The key role of the CVW being a 'bridge to the hospice' and enabling palliative care to reach out into the rural community was strongly reported by all respondents.	There is no payment for the volunteers, although a bicycle is provided to enable them to travel to see patients, along with a bicycle maintenance fee. Costs for attendance at all meetings are reimbursed. They are also provided with a kit that includes practical items such as Savlon, gloves, soap, cotton wool, bandages, plus a T-shirt that identifies them as hospice volunteers, which they are encouraged to wear while undertaking their role. Currently the annual amount per volunteer for bicycle maintenance and attendance at any updates, consumables (T-shirts, etc.), stationery and equipment for their kits (soap, gloves, etc.) is 287,960 Ugandan shillings (£93, US$148, €101).

						administration of medicine, spiritual support, basic counselling, support the patient with basic nursing care including washing,train carers in basic nursing skills including bed making, fight the fear and negative attitude**s** towards HIV/AIDS, consultations with other relevant health care workers, create links with the patients, their families and local communities.			

Jack B.A. et al.	The personal value of being a palliative care Community Volunteer Worker in Uganda: A qualitative study	2012	Uganda, East Africa	4b	**Name:** Community Volunteer Workers (CVWs)<br><br>**Number:** 32<br><br>**Description:** Members of the local community who speak the local dialect. The CVWs are selected by senior members of the local community and meet the following criteria: completed a basic education, identified as being respected and trustworthy, and available to volunteer for about 6 hours a week.<br><br>**Remuneration:** No renumeration. Cost for meeting attendance reimbursed.	**Purpose:** The aim of this study was to evaluate the motivation for becoming a volunteer and the personal impact of being a palliative care Community Volunteer Worker in Uganda.<br><br>**Duration:** No details.<br><br>**Role of CHW:** The Community Volunteer Workers augment the work of the Hospice team in identifying patients in need of palliative care, particularly in rural settings. In addition, they help to provide care for the patients, providing practical, physical, emotional and spiritual care for patients in their own homes.	**Content:** CVWs undergo a nonresidential course that covers an overview of the fundamentals of palliative care, practical aspects of home nursing care, communication skills, end-of-life care, emotional support for carers, bereavement support and ethics of palliative care.<br><br>**Duration:** 6 days.<br><br>**Person or group responsible for delivering training:** No details.<br><br>**Theories used:** No details<br><br>**Assessment of training:** No details<br><br>**Supervision:** Ongoing education and support are provided by the Hospice team, with monthly meetings being held, and contact via mobile phone for day-to-day support and advice.	There was a strong consensus amongst all the volunteers and the Hospice staff that the impetus for becoming a volunteer was based on the wish to care for the suffering in the villages. Along with the skills obtained (for example, practical, emotional, physical and spiritual support), which are reported elsewhere, being a Community Volunteer Worker resulted in a high level of personal pride. Additionally, the role impacted upon their status in their local communities and the respect that was subsequently shown to them within their communities.	The volunteers receive no payment, although they are provided with a bicycle and maintenance fund sundries (a kit that contains basic practical items such as Savlon, gloves, etc.) and a uniform T-shirt denoting their volunteer work with Hospice Africa Uganda. Active volunteers are defined as those who complete monthly reports of their activities (referrals, etc.) as well as attending the update sessions

Kang'ethe S.M. et al.	Validating that palliative care giving is a stressful occupation: The case of the Kanye community home-based care programme, Botswana	2010	Botswana, Southern Africa	3a	**Name:** Palliative caregivers<br><br>**Number:** 82<br><br>**Description:** No cadre description.<br><br>**Remuneration:** No details.	**Purpose:** This article explores the stress-related challenges facing volunteer palliative caregivers in the Kanye community home-based care (CHBC) programme.<br><br>**Duration:** No details.<br><br>**Role of CHW:** No details.	**Content:** No details.<br><br>**Duration:** No details.<br><br>**Person or group responsible for delivering training:** No details.<br><br>**Theories used:** No details.<br><br>**Assessment of training:** No details.<br><br>**Supervision:** No details.	The study found the following aspects inherent in care giving to be immensely stressful and challenging: Heavy caseload of the caregivers, ageing of the caregivers, stigma and discrimination abound in care giving, conflict between caregivers and clients, poor state of referral networks, inadequate care packages, poverty of the caregivers themselves, and inadequate food, psychological support and community support networks	No details.

Naicker S.N. et al.	Development and pilot evaluation of a home-based palliative care training and support package for young children in southern Africa	2016	South Africa, Africa	4a	**Name:** Home based care workers<br><br>**Number:** 13<br><br>**Description:** All HCBC workers had prior training in home-based care and were female. The youngest care worker was 21 and the oldest was 55 years old, with a median age of 42.<br><br>**Remuneration:** No details.	**Purpose:** The paper described the development of a palliative care package for the homecare of young children, most of whose lives are limited by untreated HIV or delayed HIV treatment, which can be tailored to suit various service delivery mechanisms. The aim was to develop a comprehensive package through research activity that sought to understand the context in which young children and their families and communities are facing life-limiting conditions and diseases with little resources.<br><br>**Duration:** No details.<br><br>**Role of CHW:** HCBC workers support caregivers to keep a child well, help caregivers to decide when and how to disclose a child's HIV status, and to show caregivers ways to ensure that the child continuously takes medication.	**Content:** Components of the training and support package for home-based carers included the following components: a Guide for Home-based Care Workers, a Training Manual for Home-based Care Workers, a Caregiver's Toolkit, a series of illustrated laminated handouts for caregivers containing important information on germs, sanitation, and disease prevention.<br><br>**Duration:** No details.<br><br>**Person or group responsible for delivering training:** No details.<br><br>**Theories used:** No details.<br><br>**Assessment of training:** Evaluation sheets designed to assess the level of learning and retention. The evaluator was made familiar with the training and support package and presented with i) a checklist to be	The HCBC workers noted a number of advantages of home-care over hospital or clinic-based care, including allowing the child to be with the family, to receive community support as well as access to traditional medicines. For the training that was designed and implemented, findings reflected that both HCBC workers and caregivers felt that the course was useful in delivering the key messages around palliative care at home for young children. Many of the older caregivers appreciated the method as a way of preserving the culture of "talking around the fire" and felt that storytelling was a much easier way of passing on learning to others and educating and entertaining children.	No details.

							used during observational visits with the HCBC worker, and ii) a semi-structured questionnaire designed to evaluate the views of the HCBC workers on their use of the training and support package subsequent to its use.<br><br>**Supervision:** Evaluator that observes the HCBC.			

Nesbit K.C. et al.	Rehabilitation training for home-based palliative care community health workers: A pilot study	2015	Malawi, Southern Africa	4a	**Name:** Community Health Workers (CHWs)<br><br>**Number:** 20<br><br>**Description:** Home-based palliative care CHWs from a rural referal hospital.<br><br>**Remuneration:** No details.	**Purpose:** The purpose of this pilot study was to evaluate a rehabilitation training programme for home-based palliative care community health workers.<br><br>**Duration:** No details.<br><br>**Role of CHW:** CHWs held the knowledge and basic skills in physical therapy for movement, comfort, recovery, and prevention. They teach proper feeding position, arm range of motion, reclined sitting, side lying for comfort, and standing up from a chair, leg range of motion, adapting the village, cane walking, cane fitting, forearm support for walking, sitting up in bed, and turning in bed.	**Content:** The training provided the CHWs with knowledge and basic skills in physical therapy for movement, comfort, recovery, and prevention.<br><br>**Duration:** 3 weeks<br><br>**Person or group responsible for delivering training:** The pre- and post-knowledge test was developed in collaboration with local experts and the Principal Investigator. Translation was provided by the home-based care nurse coordinator (Chichewa native speaker). The skills were developed by experts in rehabilitation (a licensed physical therapist with extensive experience in the setting and final-year student physical therapists). The home visit data collection checklist was developed by the Principal Investigator, the home-based care nurse coordinator and a clinical officer with	Participants (n = 20) showed significantly greater knowledge on the post-test (M = 7.35, SE = 0.406) than on the pre-test (M = 4.25, SE = 0.422, t (19) =−7.566, P = 0.000). Competency in all 22 physical therapy skills was demonstrated by 100% of the participants (n = 20). At 2-week post-training, the participants surveyed (n = 14) reported using 21 of 22 physical therapy skills and teaching all 21 of the skills used to the caregiver of the patient. This study demonstrated the knowledge and skills learned in a training programme with lecture, practice sessions and application components in a resource-limited setting.	No details.

							advanced training in palliative care.<br><br>**Theories used:** No details.<br><br>**Assessment of training:** The reliability and validity of the checklist was assessed informally through repeated administration of the checklist by several observers and consensus on the item relevance and clarity.<br><br>**Supervision:** No details.		

Nesbit K.C. et al.	Rehabilitation training for community health workers: a five-year study	2019	Malawi, Southern Africa	4a	**Name:** Community Health Workers (CHWs)<br><br>**Number:** 60<br><br>**Description:** This project sample included all the active home-based palliative care community health workers at St. Gabriel's Hospital.<br><br>**Remuneration:** No details.	**Purpose:** The purpose of this study is to evaluate the effectiveness of a rehabilitation training program for community health workers in a resource-limited setting.<br><br>**Duration:** 5 years<br><br>**Role of CHW:** CHWs held the knowledge and basic skills in physical therapy for movement, comfort, recovery, and prevention. They teach proper feeding position, arm range of motion, reclined sitting, side lying for comfort, and standing up from a chair, leg range of motion, adapting the village, cane walking, cane fitting, forearm support for walking, sitting up in bed, and turning in bed.	**Content:** The training provided the CHWs with knowledge and basic skills in physical therapy for movement, comfort, recovery, and prevention.<br><br>**Duration:** 3 weeks<br><br>**Person or group responsible for delivering training:** The pre- and post-knowledge test was developed in collaboration with local experts and the Principal Investigator. Translation was provided by the home-based care nurse coordinator (Chichewa native speaker). The skills were developed by experts in rehabilitation (a licensed physical therapist with extensive experience in the setting and final-year student physical therapists). The home visit data collection checklist was developed by the Principal Investigator, the home-based care nurse coordinator and a clinical officer with	CHWs participating in the rehabilitation training program over the past 5 years improved their knowledge, skill competency and the ability to implement what they learned to patient care in the village. These improvements are evident in the assessment of all aspects of the training – didactic, psychomotor skill and application in context. The one area that did not show improvement – 2017 knowledge – might be related to the limitations of the test instrument (the average for the pre-test was high (73%), only 5 questions on the test, clarity of the test questions)	No details.

							advanced training in palliative care.<br><br>**Theories used:** No details.<br><br>**Assessment of training:** The reliability and validity of the checklist was assessed informally through repeated administration of the checklist by several observers and consensus on the item relevance and clarity.<br><br>**Supervision:**		

Potts M. et al.	A Qualitative Evaluation of a Home-based Palliative Care Program Utilizing Community Health Workers in India	2019	India, South Asia	3b	**Name:** Community Health Workers (CHWs)<br><br>**Number:** 3<br><br>**Description:** Of the three CHWs, two had degrees in homeopathic medicine and one had a certificate in paramedics.<br><br>**Remuneration:** No details.	**Purpose:** A home-based palliative care program using CHWs to facilitate care delivery was developed to extend the reach of a cancer center's palliative care services outside of Kolkata, India. The research question guiding this qualitative study was, how feasible, useful, and acceptable was this program from the perspectives of the clinical team and CHWs who delivered the intervention? The purpose of this feasibility study was also to assess the potential for future, broader scale testing, and implementation of the palliative care program.<br><br>**Duration:** 9 weeks<br><br>**Role of CHW:** CHWs comforted patients and offered support.	**Content:** The palliative care training included (1) a few days of informal shadowing the palliative care team for 4 h a day before the formal training and (2) a formal training that included 3 h of shadowing and 4 h of didactic classroom training a day.<br><br>**Duration:** 1 week.<br><br>**Person or group responsible for delivering training:** Delivered collaboratively by SGCCRI and MUSC at the cancer center.<br><br>**Theories used:** The training was based on the Palliative Care Toolkit, a resource that offers evidence-based strategies to deliver home-based palliative care in low-resource areas.<br><br>**Assessment of training:** No details.<br><br>**Supervision:** No details.	Overall, CHWs were able to facilitate delivery of meaningful palliative care to patients and extend the reach of a cancer center's home-based palliative care program. Three major themes concerning the feasibility, usefulness, and acceptability of the home-based palliative care program emerged (1) desire and need for more CHW training, (2) tailoring of existing intervention protocols and modifying expectations of stakeholders, and (3) considerations for program sustainability. The information gained from this evaluation study and the evidence it provided suggested that this palliative care program will be feasible to implement and assess on a broader scale.	No details.

Poyhia R. et al.	Palliative Care Volunteers Have High Workload but No Burnout: A Questionnaire Survey from Tanzania.	2019	Tanzania, East Africa	4a	**Name:** Palliative Care Volunteers<br><br>**Number:** 47<br><br>**Description:** The majority (68%, 23/34) of the volunteers did not have any occupation, and only few of them reported to be a farmer, an HBC worker, a teacher, or a laboratory technician by profession. All volunteers had already worked more than three years as a volunteer. Eighty-two percent (28/34) reported that they also had previous experience of voluntary work.<br><br>**Remuneration:** Six volunteers received a monthly allowance and 16 volunteers received compensation for travel expenses. The maximum monetary support was 25,000 Tanzanian shillings (TZS) a month. The volunteers were also provided	**Purpose:** The purpose was to study the content and burden of volunteer work in the palliative home care of Ilembula District Designated Hospital (IDDH), a secondary care institution in Tanzania.<br><br>**Duration:** No details.<br><br>**Role of CHW:** Volunteer work consisted of: home visits were made by 97% of volunteers and hospital visits by 50%. The main activities of the volunteers were cleaning the patients' home (65%), assisting with visits to the doctor or hospital (56%), washing the patients (53%), dressing the patients (47%), helping with transport (42%), and preparing meals (38%). Only 21% of the volunteers had helped the patients in leisure activities. Less frequent activities of the volunteers were shopping assistance and help with medication.	**Content:** No details.<br><br>**Duration:** No details.<br><br>**Person or group responsible for delivering training:** No details.<br><br>**Theories used:** No details.<br><br>**Assessment of training:** No details.<br><br>**Supervision:** No details.	Responses to PCETK and ProQOL were received from 34 (72%) to 20 (42%) volunteers, respectively. The Kiswahili translation of ProQSL appeared to be highly reliable. On average, a volunteer worked 20 hours/ month and had 22 patients. The volunteers reported high satisfaction ratings (average 4.2, standard deviations 0.38) and had higher scores than the validation group in the compassion fatigue scale (2.42 vs. 1.55, p < 0.01) but no burnout.	Six volunteers received a monthly allowance and 16 volunteers received compensation for travel expenses. The maximum monetary support was 25,000 Tanzanian shillings (TZS) a month. The volunteers were also provided with a bicycle from the hospital, hospital resources permitting.

					with a bicycle from the hospital, hospital resources permitting.				

Taub J.M. et al.	Palliative care delivery: descriptions of community-based services in Vellore, India, and Seattle, Washington.	2019	India, South Asia and United States, North America	3b and 4b	**Name:** Village Health Workers (VHW)<br><br>**Number:** No details.<br><br>**Description:** The volunteer village health workers are nominated by community members. These volunteers are primarily women with minimal medical training, who have historically served in their village as a traditional birthing attendant.<br><br>**Remuneration:** No details.	**Purpose:**This study describes two community service programmes, one in India and one in the United States, that have created community partnerships to provide and support health promotion and social services for community-dwelling older adults with life-limiting illness. A comparison of these two programmes provides insight into how different organisations deliver palliative care within different cultural contexts and healthcare settings.<br><br>**Duration:** No details.<br><br>**Role of CHW:** The VHWs help minimise barriers related to communication, geographical location, education, or generalised mistrust and are a vital link connecting rural residents with healthcare services. The VHW works 2–3 hours each day and is responsible	**Content:** The VHW is trained to provide basic health care services for the families in the community.<br><br>**Duration:** No details.<br><br>**Person or group responsible for delivering training:** The VHW is trained by the Community Health Nursing Department, College of Nursing, CMC Vellore. Monthly in-service education programmes to upgrade VHWs' knowledge and skills are organised by the CONCH nursing team. This involves education on knowledge and skills through in-service education on various chronic illnesses and palliative care by palliative care nurses, ostomy nurses, diabetic nurse educators and other nurse specialists.<br><br>**Theories used:** No details.<br><br>**Assessment of training:** No details.	CONCH has developed a system of tiered community nursing care to provide inclusive delivery of services within an identified geographical area. This public health approach supports delivery of palliative care by a continuum from healthcare professionals to community residents; this delivery model improves access to care for older adults in rural areas and allows cost-effective delivery of healthcare and social services to a large population. CONCH health teams are able to provide direct and indirect client-focused and family-centered care in home settings, crossing socio-economic and cultural barriers to ensure quality of life for clients and their families.	Neither the CONCH Programme nor the PNA Village rely on government funding for services. CONCH is part of the Christian Medical College and is funded by client fees for services and private donations.

						for motivating families to use healthcare services, accompanying clients who need assistance in accessing hospital services, and informing the community health nurse of any health concerns clients may have.	**Supervision:** No details.		

Kumar S.K.et al.	Kerala, India: A Regional Community-Based Palliative Care Model	2007	India, South Asia	3b	**Name:** Volunteers<br><br>**Number:** Network of 4,000<br><br>**Description:** People who live in the local community, who can spare at least two hours per week to care for the sick in their area.<br><br>**Remuneration:** No details.	**Purpose:** Kerala, in India, has shown enormous progress in the area of palliative care (PC). Most of it is due to the network of community initiatives in PC in north Kerala. This network, called ''Neighborhood Network in Palliative Care,'' has more than 60 units covering a population of more than 12 million, and is probably the largest community-owned PC network in the world. The evolution and functioning of this network and the lessons learned are discussed.<br><br>**Duration:** 5 years<br><br>**Role of CHW:** The volunteers identify problems of the chronically-ill in their area and organize appropriate interventions. Volunteers make regular home visits to follow up on the patients seen by the PC team. They identify and address a variety of nonmedical issues, including	**Content:** A structured training program of interactive theory sessions plus four clinical days under supervision.<br><br>**Duration:** 16 hours<br><br>**Person or group responsible for delivering training:**<br><br>**Theories used:** No details.<br><br>**Assessment of training:** Evaluation at conclusion of training.<br><br>**Supervision:** Clinical staff during training.	The NNPC experiment has attracted attention in different ways. The estimated good coverage for PC and LTC in a ''resource-poor'' setting, the enthusiasm that has been generated in the local community, the reliance on locally generated funds, and hence the good potential for sustainability are a few among them. The partnership of the State with civil society is now seen by many as a means through which a raft of societal and political ills can be addressed. NNPC has been engaging local governments to identify and prioritize the local health needs.	No details.

						financial problems, patients in need of care, organizing program to create awareness in the community, and raising funds for PC activities. Local volunteers visit the patients at home.				
